# Mediating ferritinophagy and ferroptosis: a novel strategy against *Staphylococcus aureus* infection from a Kudzu root endophytic fungus

**DOI:** 10.1128/spectrum.03923-25

**Published:** 2026-07-02

**Authors:** Wenshu Zou, Shuyuan Cheng, Xiaoai Li, Pengjie Han, Honghong Jiao, Zhongyi Hua, Tianrui Liu

**Affiliations:** 1 Jiangxi Province Key Laboratory of Sustainable Utilization of Traditional Chinese Medicine Resources, Institute of Traditional Chinese Medicine Health Industry, China Academy of Chinese Medical Sciences, Nanchang, China; 2 Postdoctoral Fluxion Station, China Academy of Chinese Medical Sciences 71046, Beijing, China; 3 Shandong Engineering Research Center for Innovation and Application of General Technology for Separation of Natural Products, Shandong Analysis and Test Center, Qilu University of Technology (Shandong Academy of Sciences), Jinan, China; 4 School of Pharmacy, Shaanxi University of Chinese Medicine 107652, Xianyang, China; 5 School of Pharmacy, Jiangsu University 12676, Zhenjiang, China; Newcastle University, Newcastle Upon Tyne, United Kingdom

**Keywords:** *Staphylococcus aureus*, *Aspergillus aureoles *GGNSJ001, RAW 264.7 cell, ferroptosis, antibacterial

## Abstract

Serious *Staphylococcus aureus* infections pose a major global public health challenge. However, the escalating problem of antibiotic resistance has led to a progressive decline in effective therapeutic options. In this study, we demonstrated that *Aspergillus aureoles* GGNSJ001, a bioactive compound isolated from endophytic fungi of Kudzu root, effectively inhibits *S. aureus* biofilm formation *in vitro*. Further analysis revealed that the ability of *S. aureus* to adhere to and invade RAW 264.7 cells was attenuated by *A. aureoles* GGNSJ001 pre-treatment. To explore the underlying mechanism, an *S. aureus*-infected macrophage model was established, and the effect of *A. aureoles* GGNSJ001 on oxidative stress and ferroptosis was assessed. It was shown in the results that the levels of reactive oxygen species (ROS) and malondialdehyde (MDA) were elevated by *A. aureoles* GGNSJ001 treatment, while the expression of superoxide dismutase (SOD) and glutathione (GSH) was reduced. Through immunofluorescence (IF) and qRT-PCR analyses, it was further confirmed that lipid peroxidation and Fe^2+^ accumulation were promoted, and the expression of key ferroptosis-related genes *ACSL4*, *ALOX15*, *TFR1*, and *GPX4* were reversed by *A. aureoles* GGNSJ001, leading to effective control of *S. aureus* infection. Mechanistically, it was identified that ferroptosis driven by the *NCOA4–FTH1* axis constitutes the core event through which the antibacterial effects of *A. aureoles* GGNSJ001 are exerted. In summary, this study systematically illustrates how *A. aureoles* GGNSJ001 activates ferritinophagy via the *NCOA4–FTH1* pathway, thereby enhancing oxidative stress and triggering ferroptosis to clear *S. aureus*-infected macrophages. These findings offer novel mechanistic insights and a promising therapeutic strategy for combating antibiotic-resistant bacterial infections.

**IMPORTANCE:**

This work addresses the critical challenge of antibiotic-resistant *Staphylococcus aureus* infections by identifying a novel antifungal strategy. We demonstrate that a natural compound from the *Aspergillus aureoles* GGNSJ001 can clear infected immune cells by hijacking a specific cell death pathway known as ferroptosis. This represents a paradigm shift, as it targets the host's cellular machinery to combat bacteria rather than the pathogen directly, offering a promising new avenue to overcome drug resistance. The findings provide a foundational framework for developing innovative host-directed therapies against persistent bacterial infections.

## INTRODUCTION

*Staphylococcus aureus* causes a spectrum of human diseases, from minor skin infections to life-threatening bacteremia (1, 2). Its significant health burden is compounded by rising antimicrobial resistance, as seen in the high mortality of methicillin-resistant *S. aureus* infections and the emergence of vancomycin-resistant strains (3–5). A key challenge is the ability of *S. aureus* to survive within macrophages, evading immune clearance and promoting chronic, recurrent infections (6–8). This intracellular persistence, coupled with widespread antibiotic resistance, underscores the critical need for novel therapeutic strategies to combat this pathogen.

In recent years, plant-derived natural products and metabolites have revitalized scientific interest in drug discovery due to the scarcity of novel antibiotics effective against difficult-to-treat infections (9, 10). They are regarded as an important source for new-generation antibacterial agents due to their structural diversity, multi-target mechanisms of action, and relatively low toxicity. Kudzu, a traditional Chinese herb with a long history of use, stands out among medicinal plants for the anti-*S. aureus* potential of its key compounds, puerarin and daidzein (11–13). Puerarin, in particular, directly inhibits the invasion of the bacterium. This activity is complemented by its ability to attenuate subsequent tissue damage through the suppression of inflammatory signaling pathways, including NOD-like receptor protein 3 (NLRP3) and nuclear factor (NF)-κB (14, 15). Although significant progress has been made in the study of Kudzu itself and its primary active constituent Pueraria, research on its endophytic microbial communities remains in its infancy. As an important microbial resource, plant endophytes are capable of producing a variety of bioactive secondary metabolites. However, it remains unclear whether endophytic fungi derived from Kudzu root can attenuate *S. aureus*-induced macrophage inflammation, and the underlying mechanisms warrant further investigation.

Ferroptosis is a novel form of programmed cell death mediated by excessive levels of free iron and lipid peroxides (16). Ferroptosis has been closely linked to infections caused by various pathogenic bacteria, including *Mycobacterium tuberculosis*, *Pseudomonas aeruginosa*, and *S. aureus*. Current evidence indicates that ferroptosis plays a dual role during bacterial infection. On one hand, it can be induced in macrophages post-infection, facilitating bacterial dissemination and exacerbating tissue damage (17). On the other hand, ferroptosis may also be activated as a host defense mechanism. This process promotes iron accumulation and elevated lipid peroxidation in macrophages, leading to the death of infected cells and thereby clearing intracellular bacteria, restricting pathogen proliferation, and mitigating excessive inflammation (18–20). RAW 264.7 macrophages, as key immune effector cells, represent an important line of defense; however, whether they undergo ferroptosis upon *S. aureus* infection remains unclear, and the underlying mechanisms await further elucidation. Additionally, the potential of *A. aureoles* GGNSJ001 to mitigate *S. aureus* infection through the regulation of ferroptosis has not been established.

Here, we systematically evaluated the efficacy of *A. aureoles* GGNSJ001 against *S. aureus* infections through a series of *in vitro* assays and elucidated its underlying mechanisms. We demonstrated that *A. aureoles* GGNSJ001 pretreatment significantly suppressed the adhesion to and invasion of RAW 264.7 cells by *A. aureoles* GGNSJ001. Moreover, *A. aureoles* GGNSJ001 enhanced oxidative stress and triggered ferroptosis, leading to the elimination of infected macrophages. Mechanistic studies revealed that *A. aureoles* GGNSJ001 activates *NCOA4–FTH1*-mediated ferritinophagy, thereby promoting ferroptosis and conferring protection against *S. aureus* infection in macrophages. These findings highlight ferroptosis as a promising therapeutic target and provide new insights into potential treatment strategies for bacterial infection-related diseases.

## MATERIALS AND METHODS

### Isolation, purification, and identification of endophytic fungi from Kudzu root

A total of 1 g of surface-sterilized Kudzu root was homogenized in a sterile mortar with 5 mL of sterile water. The homogenate was serially diluted and plated onto PDA plate (Solarbio, Beijing, China), followed by incubation at 37 °C. Plates were examined daily for microbial growth. Distinct fungal hyphae were subcultured onto fresh media repeatedly until pure isolates were obtained. Fungal biomass was pelleted by centrifugation, and genomic DNA was amplified directly using a fungal extraction and amplification kit (Thermo Fisher, USA). Sanger sequencing of the amplified products was conducted by BGI.

The purified *A. aureoles* GGNSJ001 was inoculated into PDA liquid medium and cultured at 37°C with shaking at 200 rpm for 72 h. The fermentation broth was filtered through a 0.22-μm sterile membrane to remove mycelia and obtain cell-free metabolites. The initial inoculum concentration of the fungus was 1 × 10⁶ CFU/mL. All other tested endophytic fungi were adjusted to OD_600_ = 0.6 before fermentation to ensure consistent biomass.

### *Staphylococcus aureus* growth conditions

*S. aureus* ATCC 29213 was purchased from National Center for Medical Culture Collections (Beijing, China). Stock cultures were maintained at −80°C in LB (Solarbio), 1% tryptone, 0.5% yeast extract, and 1% sodium chloride) supplemented with 20% glycerol. The strains were resuscitated by inoculating LB broth and shaking at 37°C overnight to achieve a final concentration of ~10^9^ CFU/mL.

### Determination of MIC

The MIC of the cell-free metabolites of *A. aureoles* GGNSJ001 against *S. aureus* was determined by a two-fold broth microdilution assay in TSB. The metabolite was serially diluted in a 96-well plate, followed by the addition of a standardized bacterial inoculum (1 × 10^6^ CFU/mL). After 24-h incubation at 37 °C, OD_600_ was measured. The MIC was defined as the lowest concentration that reduced bacterial growth by ≥90% compared with the blank control. The MIC value was determined as 500 μg/mL based on three independent experiments.

### *S. aureus* bacteriostatic circle assay

The antibacterial activity of the test compound against *S. aureus* was assessed using the agar well diffusion method. A bacterial suspension was evenly swabbed onto LB plates. Wells were punched into the agar and filled with the cell-free metabolites of endophytic fungi, negative control (sterile LB medium), and positive control (vancomycin, 50 μg/mL). After pre-diffusion at room temperature for 30 min, the plates were incubated at 37°C for 18–24 h. The antibacterial activity was quantified by measuring the diameter of the bacteriostatic circles in millimeters, with results presented as the mean of triplicate experiments.

### Cell counts in biofilm

For biofilm assays, overnight cultures were diluted to ~1 × 10^7^ CFU/mL in LB broth and incubated with or without *A. aureoles* GGNSJ001 at 1/2× MIC (250 μg/mL), 1× MIC (500 μg/mL), 2× MIC (1,000 μg/mL) for up to 72 h at 37°C. All experiments used 0.22-μm filtered cell-free metabolites without fungal mycelia.

At designated time points (24, 48, and 72 h), biofilms were washed with PBS to remove planktonic cells, detached using 0.1% trypsin (BasalMedia, Shanghai, China), and quantified via viable plate counting on LB agar.

Biofilms were lysed with 0.1% trypsin, which specifically lyses *S. aureus* but not fungi. A group containing only fungal metabolites was included as a control to exclude biofilm formation from fungal components. Only *S. aureus* colonies were counted to avoid interference from fungal biomass.

### Bacterial growth measurements

To assess the growth kinetics, bacteria were first grown to the early exponential phase. Following this, they were standardized to 1 × 10^7^ CFU/mL in LB medium containing *A. aureoles* GGNSJ001 at sub-inhibitory (1/2× MIC), inhibitory (MIC), and supra-inhibitory (2× MIC) concentrations; an LB culture without the compound was used as the control. Subsequently, 200 μL of each suspension was added to the wells of a 96-well microtiter plate. The plate was incubated under static conditions at 37°C, and the growth was quantified by measuring the OD_600_ at 24-h intervals over a 72 h period.

### RAW 264.7 line

RAW 264.7 cells were maintained in culture in Dulbecco’s modified Eagle media (DMEM, BasalMedia) supplemented with 1% penicillin-streptomycin liquid (Solarbio), 10% fetal bovine serum (FBS, Gibico, USA). RAW 264.7 cells were seeded at a density of approximately 1 × 10^5^ cells in per tissue culture plate (Corning), containing 2 mL of complete medium.

### Adhesion in RAW 264.7 cells

Bacterial adhesion was quantified as follows: a 200-μL aliquot of bacterial culture was inoculated into each well of a 96-well plate (Corning) and incubated for 3 h. To remove non-adherent bacteria, the supernatant was discarded, and the monolayers were washed five times with PBS. Subsequently, the adherent bacteria were lysed by adding 200 μL of 1% Triton X-100 (Solarbio) in PBS for 10 min. The lysates were then collected, serially diluted, and plated on LB agar. After 24 h of incubation at 37°C, the colony-forming units (CFUs) were counted to determine the number of adherent bacteria.

Adhesion (%) = (Counts of released bacteria after Triton treatment / Counts of added bacteria) ×100.

### Invasion in RAW 264.7 cells

RAW 264.7 cells in a 24-well plate (Corning) were inoculated with a bacterial suspension and incubated for 3 h at 37°C with 5% CO_2_. To eliminate non-internalized bacteria, gentamicin was added to a final concentration of 100 mg/L for 30 min. Following this treatment, the monolayers were thoroughly washed with PBS. The RAW 264.7 cells were then lysed using sterile distilled water. The resulting lysates were diluted and plated on LB agar to determine the number of viable intracellular *S. aureus* colonies (CFUs) after 24 h of incubation.

### CCK8

The viability of RAW 264.7 cells was evaluated by the CCK-8 assay, which measures the metabolic activity of NAD(*P*)H-dependent cellular oxidoreductases. Cells were plated at 1 × 10^3^ cells/well in a 96-well plate and treated accordingly. The CCK-8 reagent (Solarbio), which is reduced by these enzymes in viable cells to form an orange-colored formazan dye, was added and incubated. The absorbance of this water-soluble formazan at 450 nm was quantified using a microplate reader, and the results were used to calculate the percentage of cell viability.

### ELISA

RAW 264.7 cells were seeded in 96-well plates at a density of 1 × 10^5^ cells per well and cultured for 24 h to reach confluency. The cells were first primed with *S. aureus* at a MOI of 10 for 3 h. Without removing the media, the cultures were then stimulated with varying concentrations (1/2×, 1×, and 2× MIC) of *A. aureoles* GGNSJ001 for an additional 24 h. Following incubation, the culture supernatants were collected, and the levels of *IL-6*, *IL-1β*, *IL-4*, *IL-10*, and *TNF-α* were quantified using specific ELISA kits (Cloud-Clone Corp, Wuhan, China) in strict accordance with the manufacturer’s protocols. Cell culture supernatants were subjected to total protein concentration measurement using the BCA (Beyotime, Shanghai, China) kit before use, and then adjusted to a uniform total protein concentration with PBS. All data were normalized to total cellular protein concentration to eliminate variation caused by differences in cell number.

### ROS, MDA, SOD, and GSH assay

The levels of key oxidative stress markers were assessed using commercial kits according to the manufacturers' protocols. Intracellular ROS were detected using a Reactive Oxygen Species Assay Kit (Biolab, Beijing, China). The concentration of MDA, a lipid peroxidation end-product, was measured with an MDA assay kit (Beyotime, Shanghai, China). Additionally, the activity of SOD and the content of reduced GSH were determined using their respective assay kits (both from Beyotime, Shanghai, China). For all samples, total cellular protein concentration was determined using a BCA assay kit prior to detection. All results were normalized to total cellular protein concentration to ensure comparability between samples.

### C11 BODIPY (581/591) assay

Lipid peroxidation was measured with the C11 BODIPY 581/591 probe (Beyotime). After the indicated treatments, RAW 264.7 cells were incubated with the probe for 30 min at 37 °C. Following washes, fluorescence was detected, and the ratio of green (oxidized) to red (reduced) signal was calculated to quantify peroxidation levels, as per the kit protocol.

### Fe^2+^ assay

Intracellular Fe^2+^ levels were measured using the FerroOrange fluorescent probe (Abmole, USA). Briefly, after the indicated treatments, RAW 264.7 cells were washed twice with Hank’s Balanced Salt Solution (HBSS) and subsequently incubated with 1 μM FerroOrange in HBSS at 37 °C for 30 min. Following incubation, the cells were washed again to remove excess probe. Fluorescence imaging was performed using a confocal fluorescence microscope with an excitation/emission wavelength of 580 nm.

### JC-1 assay

ΔΨm was measured in treated RAW 264.7 cells using a JC-1 Mitochondrial Membrane Potential Assay Kit (Solarbio). After staining and washing as per the instructions, fluorescence was imaged. JC-1 aggregates (red) indicate polarized mitochondria, while cytosolic monomers (green) indicate depolarization. The degree of depolarization was quantified as the green/red fluorescence intensity ratio using ImageJ.

### Q-RT-PCR assay

Total RNA was extracted from RAW 264.7 cells with TRIzo reagent (Invitrogen, USA). According to the manufacturers’ protocols, complementary DNA (cDNA) was then synthesized from the extracted RNA using a Takara Reverse Transcription Kit (Takara, Japan). Subsequent q-RT-PCR analyses were conducted with TB Green Premix EX Taq II (Takara) on a QuantStudio system. The relative mRNA expression levels were calculated using the *2−ΔΔCt* method after normalization to *β-actin*. All primer sequences are listed in Table S1.

### IF assay

RAW 264.7 cells, post-incubation under the specified conditions, were prepared for immunofluorescence staining. Briefly, cells were washed with PBS and fixed in 4% paraformaldehyde for 10 min. After permeabilization and blocking with 5% BSA (Beyotime) for 30 min to prevent non-specific binding, the cells were probed with the respective primary antibodies at 4°C overnight. The following day, after thorough washes, antigen-antibody complexes were labeled with appropriate fluorescent secondary antibodies for 1 h. Nuclei were visualized with DAPI (Beyotime) staining. A confocal fluorescence microscope was employed to acquire high-resolution z-stack images. The primary and secondary antibodies utilized in this study are cataloged in Table S2.

### Statistical analysis

All experiments were repeated three times with duplicate samples. These data were analyzed by one-way analysis of variance (ANOVA). Distribution comparison test was used to compare the mean values. A value of *P* less than 0.05 was considered statistically significant.

## RESULTS

### Isolate GGNSJ001 was identified as *A. aureoles*

The isolate GGNSJ001 was grown on PDA plates for 9 days. The cultural and morphological characteristics are shown in Fig. S1. Phylogenetic analysis of GGNSJ001 was performed using the neighbor-joining (N-J) method. The neighbor-joining tree was constructed based on the rDNA-ITS sequences. The numbers at nodes represent the percentage of their occurrence in 1,000 bootstrap replicates. The results indicated that the isolate GGNSJ001 was identified as *A. aureoles*.

### *A. aureoles* GGNSJ001 inhibited the growth, reduced adhesion, and reduced invasion of *S. aureus in vitro*

**Fig 1:**
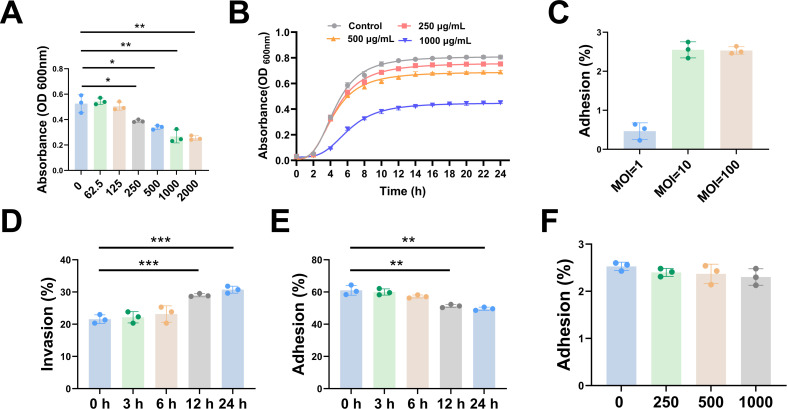
*A. aureoles* GGNSJ001 alters the physiology of *S. aureus*. Killing test (**A**) and growth curve (**B**) of *S. aureus* exposed to different concentrations of *A. aureoles* GGNSJ001. The effect of bacterial concentration on the adhesion of *S. aureus* to RAW 264.7 cells (**C**). RAW 264.7 cell adhesion (**D**) and invasion (**E**) of *A. aureoles* GGNSJ001-exposed *S. aureus* compared with the control culture. The effect of co-incubation with *A. aureoles* GGNSJ001 on *S. aureus* adhesion to RAW 264.7 cells (**F**). The error bars represent the standard error of the mean. *P < 0.05, **P < 0.01, and ***P < 0.001. All data are representative of three independent biological experiments performed in triplicates.

To evaluate the anti-*S*. *aureus* activity of GGNSJ001, we performed zone-of-inhibition assays. *A. aureoles* GGNSJ001 effectively inhibited the growth of *S. aureus in vitro* with a 26-mm inhibition zone (Table S3). The MIC of *A. aureoles* GGNSJ001 against *S. aureus* was determined to be 500 μg/mL (Table S3). This MIC value served as a key reference for subsequent experiments assessing its antibiofilm activity. Based on these findings, concentrations of 250 μg/mL (1/2× MIC), 500 μg/mL (1× MIC), and 1,000 μg/mL (2× MIC) were selected for subsequent experiments. Treatment with 250 μg/mL (1/2× MIC) or higher concentrations for 24 h significantly reduced bacterial viability (*P <* 0.05) (Fig. 1A).

The adhesion of *S. aureus* using the RAW 264.7 macrophage cell model. Fig. 1C shows the adhesion of *S. aureus* to RAW 264.7 cells depending on the multiplicity of infection‌ (MOI) 1:10, 1:50, and 1:100. Adhesion was 2.5% in the three different conditions studied, so no significant differences in adhesion were observed between the different cell/bacteria ratios evaluated (Fig. 1C). Therefore, an MOI of 1:10 was selected for subsequent experiments to evaluate the effect of *A. aureoles* GGNSJ001 on *S. aureus* adhesion to RAW 264.7 cells.

*S. aureus* pre-treated with *A. aureoles* GGNSJ001 for 12 and 24 h showed significantly reduced adhesion to RAW 264.7 cells compared with the control (Fig. 1D, *P <* 0.05). In terms of invasion, the ability of *S. aureus* exposed to *A. aureoles* GGNSJ001 to invade RAW 264.7 cells was significantly reduced at both 12 and 24 h pre-treatment (Fig. 1E, *P* < 0.05). In competition assays, in which *S. aureus* was co-incubated simultaneously with *A. aureoles* GGNSJ001, no significant differences in bacterial adhesion were detected relative to the control (Fig. 1F).

These findings suggested that *A. aureoles* GGNSJ001 inhibited the growth, reduced the adhesion, and reduced the invasion of *S. aureus*. The anti-adhesive mechanism of *A. aureoles* GGNSJ001 is not attributable to non-specific steric hindrance. In conclusion, *A. aureoles* GGNSJ001 modulates key physiological processes that underpin the pathogenicity of *S. aureus*.

### *A. aureoles* GGNSJ001 inhibits the inflammation induced by *S. aureus* in RAW 264.7 cells

**Fig 2:**
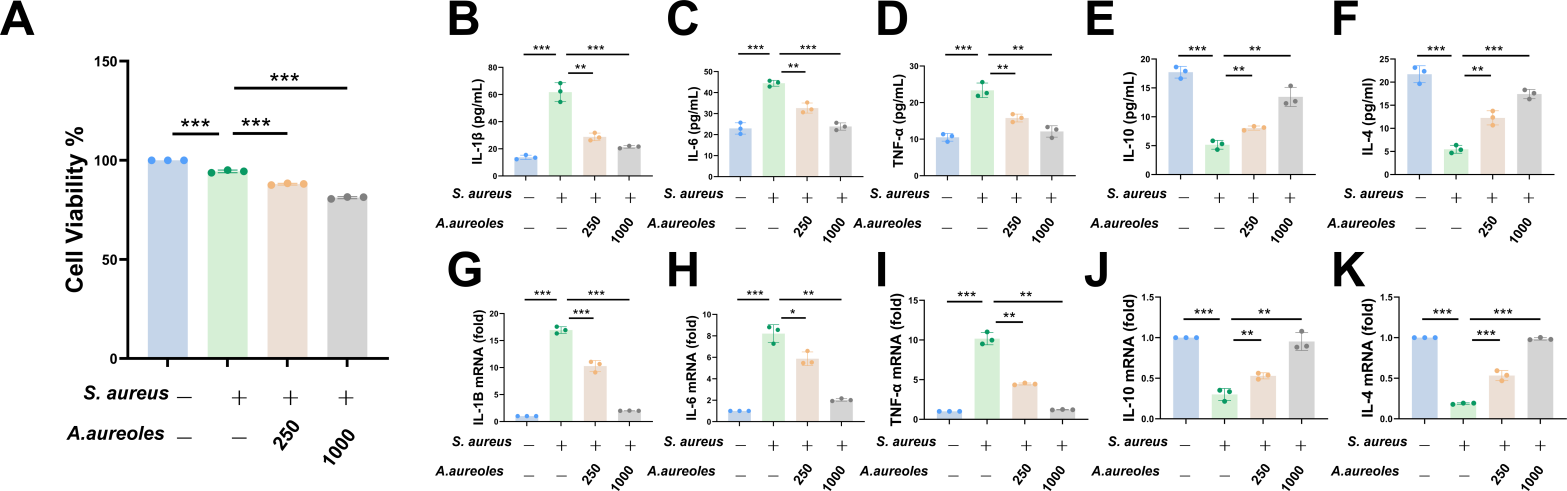
*A. aureoles* GGNSJ001 protects RAW 264.7 cells against *S. aureus*-triggered inflammation. Cell viability was measured by CCK8 staining (**A**). ELISA was used to detect the expression levels of inflammatory factors *IL-1β*, *IL-6*, *TNF-α*, *IL-10,* and *IL-4* proteins (**B–F**). The relative mRNA levels of *IL-1β*, *IL-6*, *TNF-α*, *IL-10*, and *IL-4* were assessed by qRT-PCR (**G–K**). The error bars represent the standard error of the mean. *P < 0.05, **P < 0.01, and ***P < 0.001. All data are representative of three independent biological experiments performed in triplicates.

CCK8 results showed that cell viability was decreased after *S. aureus* infection, while *A. aureoles* GGNSJ001 further reduced the cell viability in a dose-dependent manner, suggesting induction of a regulated cell death pathway (Fig. 2A). ELISA and qRT-PCR results showed that *A. aureoles* GGNSJ001 significantly reduced pro-inflammatory cytokines *IL-1β*, *IL-6*, and *TNF-α* and increased anti-inflammatory cytokines *IL-10* and *IL-4* (Fig. 2B through K). These findings indicated that *A. aureoles* GGNSJ001 effectively attenuated *S. aureus*-induced inflammatory damage in RAW 264.7 cells *in vitro*.

### *A. aureoles* GGNSJ001 promoted oxidative stress in *S. aureus*-infected RAW 264.7 cells

**Fig 3:**
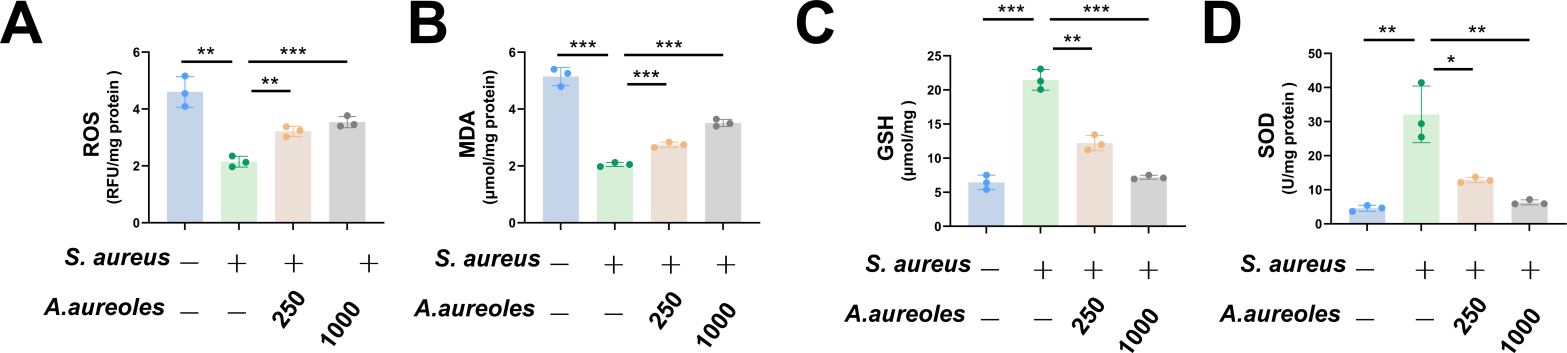
*A. aureoles* GGNSJ001 protects RAW 264.7 cells against *S. aureus* infection by enhancing oxidative stress. RAW 264.7 cells were treated with *S. aureus* (MOI = 10) for the indicated time. *A. aureoles* GGNSJ001 was added to RAW 264.7 cell culture medium before and after *S. aureus* infection for the indicated time. Effect of *A. aureoles* GGNSJ001 on the expression levels of ROS (**A**), MDA (**B**), SOD (**C**),and GSH (**D**) in RAW 264.7 cells induced by *S. aureus*. The error bars represent the standard error of the mean. *P < 0.05, **P < 0.01, and ***P < 0.001. All data are representative of three independent biological experiments performed in triplicates.

*S. aureus* infection significantly reduced ROS and MDA levels in RAW 264.7 cells, while concurrently elevating SOD and GSH levels. *A. aureoles* GGNSJ001 treatment dose-dependently restored the ROS and MDA levels and reduced the SOD and GSH levels in *S. aureus*-infected cells (Fig. 3A through D, *P <* 0.05). These findings indicated that *A. aureoles* GGNSJ001 enhanced the *S. aureus*-induced oxidative burst in RAW 264.7 cells, which might contribute to its role in promoting the oxidative stress-mediated clearance of the bacteria.

### *A. aureoles* GGNSJ001 activated ferroptosis in infected macrophages

**Fig 4:**
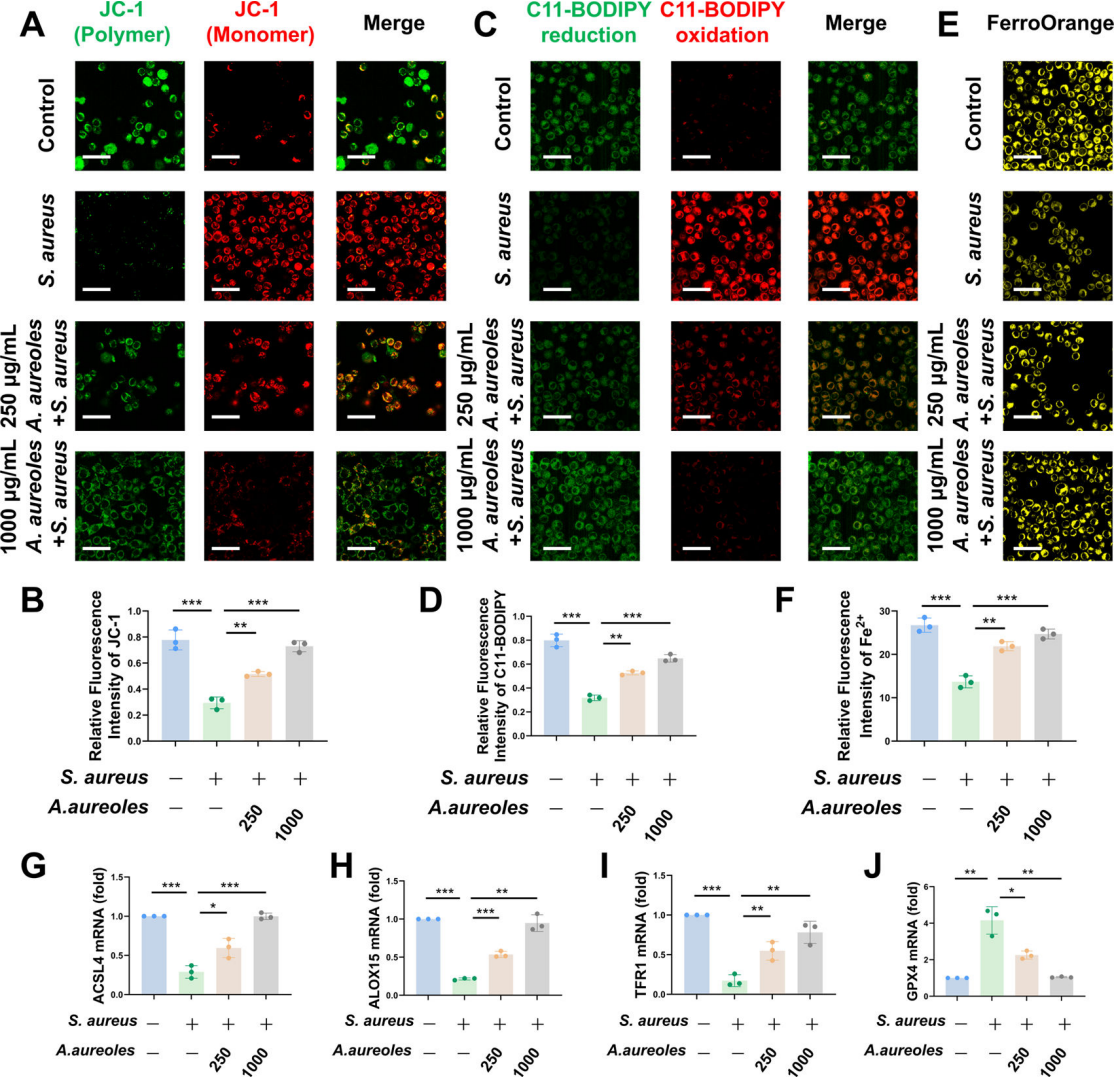
*A. aureoles* GGNSJ001 protects RAW 264.7 cells against *S. aureus*-triggered ferroptosis. RAW 264.7 cells were treated with *S. aureus* (MOI = 10) for the indicated time. *A. aureoles* GGNSJ001 was added to RAW 264.7 cell culture medium before and after *S. aureus* infection for the indicated time. JC-1 staining assessed membrane potential (**A and B**). Lipid peroxide production was assessed by BODIPY 581/591 C11 staining (**C and D**). Fe^2+^ levels were assessed by FerroOrange staining. (**G–J**) The relative mRNA levels of *ACSL4* (**G**), *ALOX15* (**H**), *TFR1* (**J**), and *GPX4* (**J**) were assessed by q-RT-PCR (**E and F**). The error bars represent the standard error of the mean. *P < 0.05, **P < 0.01, and ***P < 0.001. All data are representative of three independent biological experiments performed in triplicates.

*S. aureus*-infected RAW 264.7 cells showed a significant reduction in ΔΨm compared with the control group (Fig. 4A and B, *P <* 0.05). Treatment with *A. aureoles* GGNSJ001 markedly attenuated this loss of mitochondrial membrane potential (Fig. 4A and B, *P <* 0.05). We next measured lipid peroxidation levels using C11-BODIPY by IF. The results confirmed that *S. aureus* infection significantly decreased lipid peroxidation in RAW 264.7 cells (Fig. 4C and D, *P <* 0.05). Similarly, intracellular Fe^2+^ levels, detected with a FerroOrange probe, were also reduced upon infection (Fig. 4E and F). Importantly, *A. aureoles* GGNSJ001 treatment effectively enhanced both *S. aureus*-induced lipid peroxidation and Fe^2+^ accumulation (Fig. 4E and F). At the transcriptional level, pro-ferroptotic genes *ACSL4*, *ALOX15*, and *TFR1* were upregulated, and *GPX4* was downregulated (Fig. 4G through J, *P <* 0.05).

To verify the role of the NCOA4–FTH1 pathway, ferrostatin-1 (ferroptosis inhibitor) and NCOA4 siRNA were used. Inhibition of ferroptosis or knockdown of NCOA4 significantly reversed the effects of *A. aureoles* GGNSJ001 on ferritinophagy, lipid peroxidation, and intracellular *S. aureus* clearance.

### *A. aureoles* GGNSJ001 activates ferritinophagy via NCOA4–FTH1 axis

**Fig 5:**
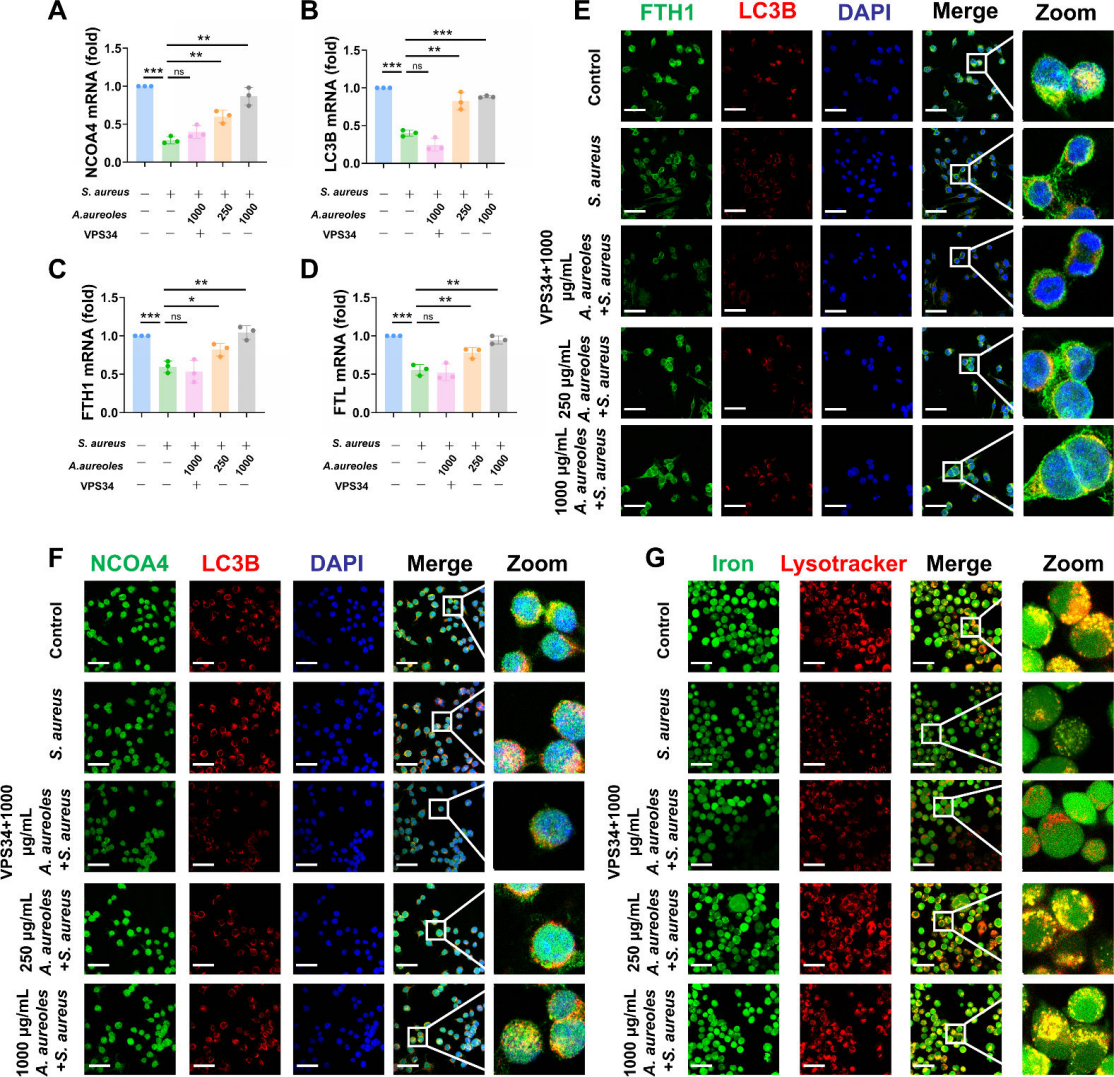
*A. aureoles* GGNSJ001 protects RAW 264.7 cells against *S. aureus*-triggered ferritinophagy. RAW 264.7 cells were treated with *S. aureus* (MOI = 10) for the indicated time. VPS34 and *A. aureoles* GGNSJ001 were added to RAW 264.7 cell culture medium before and after *S. aureus* infection for the indicated time. The relative mRNA levels of *NCOA4* (**A**), *LC3B* (**B**)*, FTH1* (**C**), and *FTL* (**D**) were assessed by q-RT-PCR. RAW 264.7 cells were subjected to immunofluorescence microscopy with antibodies against *FTH1* (green) /*LC3B* (red) (**E**) and *NCOA4* (green) /*LC3B* (red) (**F**). The content of Fe^2+^ in lysosomes was determined using Calcein-AM (green) and Lyso-Tracker Red probes (**G**). The error bars represent the standard error of the mean. *P < 0.05, **P < 0.01, and ***P < 0.001. All data are representative of three independent biological experiments performed in triplicates.

To elucidate the mechanism by which *A. aureoles* GGNSJ001 inhibits *S. aureus*-induced ferroptosis in RAW 264.7 cells, we focused on the role of cellular iron metabolism. Iron ions are not only essential for *S. aureus* survival and virulence but also serve as a central trigger of ferroptosis. We therefore hypothesized that ferritinophagy, a selective autophagic process, mediates ferroptosis in this context. This process involves the cargo receptor NCOA4, which binds to the ferritin subunit FTH1 and targets the iron-loaded ferritin complex to autolysosomes for degradation. To test this hypothesis, we first examined the mRNA expression of key ferritinophagy-related genes (*NCOA4*, *FTH1*, *FTL*, and *LC3B*) in *S. aureus*-infected RAW 264.7 cells with or without *A. aureoles* GGNSJ001 treatment. Q-RT-PCR results showed that following *S. aureus* stimulation, mRNA expression levels of *NCOA4*, *FTH1*, *FTL*, and *LC3B* were significantly decreased in RAW 264.7 cells, consistent with the findings in a previous study (9) (Fig. 5A through D, *P <* 0.05). *A. aureoles* GGNSJ001 treatment effectively reversed these expression changes (Fig. 5A through D, *P <* 0.05).

We further assessed the protein interaction by IF, analyzing the colocalization of NCOA4 with LC3B and FTH1 with LC3B. Consistent with the transcriptional results, *A. aureoles* GGNSJ001 significantly enhanced the colocalization of both FTH1-LC3B (Fig. 5E) and NCOA4-LC3B (Fig. 5F) in *S. aureus*-stimulated RAW 264.7 cells.

Iron-catalyzed lipid peroxidation constitutes the core process of ferroptosis; however, its precise subcellular site of action remains incompletely understood. Recent evidence suggests that lysosomal iron pools can trigger ferroptotic cell death (21). To visualize the subcellular distribution of Fe²^+^, we co-loaded RAW 264.7 cells with Calcein-AM (a probe for labile Fe^2+^) (22) and Lyso-Tracker Red (a lysosomal marker). Imaging revealed a decrease in the colocalization of lysosomes with Fe²^+^ in *S. aureus*-infected cells, a phenomenon that was mitigated by treatment with *A. aureoles* GGNSJ001 (Fig. 5G). To further validate whether *A. aureoles* GGNSJ001 suppresses *S. aureus*-induced impairment of NCOA4-FTH1 ferritinophagy, we used VPS34 (CAS No. 1383716-33-3, Abmole, USA), a specific inhibitor previously reported to effectively block NCOA4-FTH1 ferritinophagy (selective VPS34 inhibitor blocks autophagy and uncovers a role for NCOA4 in ferritin degradation and iron homeostasis *in vivo*). RAW 264.7 cells were pre-treated with 1 μM VPS34 for 2 h prior to *S. aureus* infection. qPCR results showed that VPS34 pre-treatment altered the mRNA levels of *NCOA4*, *FTH1*, *FTL*, and *LC3B* induced by *A. aureoles* GGNSJ001 in *S. aureus*-infected RAW 264.7 cells (Fig. 5A through D, *P <* 0.05). Immunofluorescence analysis further demonstrated that after VPS34 pre-treatment, *A. aureoles* GGNSJ001 failed to promote the colocalization of *FTH1* with *LC3B*, *NCOA4* with *LC3B*, or lysosomes with Fe^2+^ (Fig. 5E through G). These findings support that *A. aureoles* GGNSJ001 induces ferroptosis by activating the NCOA4-FTH1 ferritinophagy axis in *S. aureus*-infected RAW 264.7 cells.

## DISCUSSION

The persistence of *S. aureus* within host cells and antibiotic resistance highlight the need for new therapies. We found that *A. aureoles* GGNSJ001 inhibits adhesion and invasion and induces ferroptosis in infected macrophages to clear intracellular bacteria. The active components in *A. aureoles* GGNSJ001 are mainly sesquiterpenes, phenolic acids, and alkaloids, which are widely reported to exert antibacterial activity and regulate ferroptosis. Further purification and identification of single active compounds will be performed using LC-MS/MS.

Although the MIC of the crude metabolite (500 μg/mL) appears relatively high, this is common for natural product mixtures. CCK8 assays confirmed that 2× MIC (1,000 μg/mL) showed no obvious cytotoxicity toward RAW 264.7 cells. Further formulation optimization (e.g., nano-delivery systems) will improve local bioavailability for *in vivo* applications.

The attachment and biofilm-forming capabilities of bacterial pathogens play a pivotal role in their pathogenicity (23). Consequently, these traits rank among the most widely investigated aspects of bacterial physiology. Consistently, we demonstrated that *A. aureoles* GGNSJ001 potently impaired the adhesive and invasive functions of *S. aureus* against RAW 264.7 cells. Upon pathogen invasion, a robust inflammatory response is typically triggered in the host. This recognition process rapidly activates macrophages through pattern recognition receptors, leading to the release of pro-inflammatory cytokines, such as *TNF-α*, *IL-1β*, and *IL-6*, while concurrently suppressing anti-inflammatory factors, including *IL-10* and *IL-4* (24, 25). In this study, *A. aureoles* GGNSJ001 significantly suppressed the expression levels of proinflammatory factors *TNF-α*, *IL-1β*, and *IL-6* while increasing the expression levels of anti-inflammatory factors *IL-10* and *IL-4*, demonstrating that AA can inhibit the inflammatory response induced by *S. aureus* challenge in RAW 264.7 cells. In line with this, *A. aureoles* GGNSJ001 has been shown to decrease the adhesive and invasive functions of *S. aureus* and reduce inflammatory responses in RAW 264.7 cells.

Ferroptosis serves as a key source of damage-associated molecular patterns (DAMPs), which are critical for initiating and exacerbating inflammatory responses (26). Although several studies have demonstrated that suppressing oxidative stress and ferroptosis in mammary epithelial cells can effectively curb *S. aureus* infection (27, 28), this approach may not apply to bacteria that have evaded into macrophages. These intracellular pathogens exploit the host cell as a sanctuary, shielding them from both antibiotic treatment and immune attacks. Consequently, selectively inducing ferroptosis in infected macrophages may represent a novel host-directed therapeutic strategy to eliminate this intracellular bacterial reservoir. Our study also found that *A. aureoles* GGNSJ001 significantly elevated both *S. aureus*-induced oxidative stress levels and ferroptosis levels in RAW 264.7 cells. Thus, our findings reveal a therapeutic potential for *A. aureoles* GGNSJ001 in mitigating inflammatory responses in infected hosts by promoting ferroptosis.

Iron ions catalyze the production of substantial reactive oxygen species through the Fenton reaction, subsequently triggering lethal lipid peroxidation that collectively induces ferroptosis in immune cells, thereby facilitating bacterial escape from host immune defenses (29). Restricting bacterial access to iron has been widely recognized as an effective antibacterial strategy (30). During *S. aureus* infection, macrophages accelerate the recycling and storage of iron from senescent red blood cells, while the liver enhances the synthesis of ferritin to sequester iron within secure molecular “depots” (31). This study found that free Fe²^+^ levels were significantly reduced in *S. aureus*-infected RAW 264.7 cells. Further mechanistic investigation revealed that *A. aureoles* GGNSJ001 effectively inhibits *S. aureus* infection by activating NCOA4–FTH1 axis-mediated ferritinophagy. These results collectively demonstrate that *A. aureoles* GGNSJ001-promoted ferroptosis is closely associated with the ferritinophagy pathway regulated by the NCOA4–FTH1 axis.

This study provides a host-directed therapeutic strategy against intracellular *S. aureus* infection. By triggering ferritinophagy and ferroptosis in infected macrophages, *A. aureoles* GGNSJ001 effectively eliminates intracellular bacteria. Future studies will include *in vivo* efficacy, pharmacokinetics, and safety evaluations to support clinical translation.

### Conclusion

This study reveals that *A. aureoles* GGNSJ001 combats *S. aureus* infection by modulating oxidative stress and ferroptosis, presenting a novel strategy to overcome bacterial resistance. Future work will validate its efficacy *in vivo* and identify its precise molecular targets within the ferroptosis pathway, laying the groundwork for developing new anti-infective therapies.

## Data Availability

The ITS rRNA gene sequences of strain GGNSJ001 are uploaded in GenBank with the accession number PX664345.

## SUPPLEMENTAL MATERIAL

Supplemental material spectrum.03923-25-s0001.docx Fig. S1; Tables S1 to S3.

ASM does not own the copyrights to Supplemental Material that may be linked to, or accessed through, an article. The authors have granted ASM a non-exclusive, world-wide license to publish the Supplemental Material files. Please contact the corresponding author directly for reuse.
