# Supplementary material for: Mediating ferritinophagy and ferroptosis: a novel strategy against Staphylococcus aureus infection from a Kudzu root endophytic fungus
Source: Microbiol Spectr. 2026 Jul 2;14(8):e03923-25. doi: 10.1128/spectrum.03923-25 (PMC13435794; doi:10.1128/spectrum.03923-25)
Supplement: Supplementary file 1 [file spectrum.03923-25-s0001.docx]

Supplementary Material

**Supplementary figure S1. Isolate GGNSJ001 was identified as *A. aureoles*.** Isolate GGNSJ001 grown on PDA for 9 days (a-e). a: Front, b: Back; c: Conidial head; d: Conidiophore; e: conidia. A neighbor-joining tree was constructed based on the rDNA-ITS sequences (f). The number at nodes represent the percentage of their occurrence in 1,000 bootstrap replicates. GenBank accession numbers corresponding species names are as follows: PX664345 (Aspergillus aureoles GGNSJ001), OM436890.1 (Aspergillus aureoles BLH 31), KM613144.1 (Aspergillus aureoles URM7034), EF669945.1 (Aspergillus aureoles NRRL 20643), EF669612.1 (Aspergillus neoniveus NRRL 5299), NR 137476.1 (Aspergillus niveus NRRL 5505), FJ531213.1 (Aspergillus terreus IBT 6450), MT306007.1 (Aspergillus_oryzae JR), MH864240.1 MH864240.1 ( Penicillium citrinum CBS:126809 ).

**Supplementary table S1. Gene-Specific Oligonucleotide Primers Used for q-RT-PCR**

| **Gene** | **Serial number** | **Primer sequence (**5′-3′**)** |
| --- | --- | --- |
| *ACSL4* F | NM_001033600.1 | CCCCTTCAGACATGGCCATT |
| *ACSL4* R |  | TACGTTCACACTGGCCTGTC |
| *ALOX15* F | NM_009660.3 | GTTCAGGAACCACAGGGAGG |
| *ALOX15* R |  | GTCAGAGATACTGGTCGCCG |
| *GPX4* F | NM_001037741.4 | AAAGTCCTAGGAAACGCCCG |
| *GPX4* R |  | CTGTTGCAGTACTGGGGAGG |
| *TFR1* F | NM_001357298.1 | TATCTTCTGGGGCTCTGGCT |
| *TFR1* R |  | AGAGAGGGCATTTGCGACTC |
| *NCOA4* F | NM_001033988.3 | AGATACATCTGCTCTGCGCC |
| *NCOA4* R |  | GCCACTGGATGCTGACTTCT |
| *FTH1* F | NM_010239.2 | AGACCGTGATGACTGGGAGA |
| *FTH1* R |  | GCCAGTTTGTGCAGTTCCAG |
| *FTL* F | NM_000146.4 | CAGGCCTCCTACACCTACCT |
| *FTL* R |  | TGCATCTTCAGGAGACGCTC |
| *LC3B* F | NM_001364358.1 | CTGAGCAGAGGGAAAAGGGG |
| *LC3B* R |  | CCAGGAGGAAGAAGGCTTGG |
| *β-actin* F | XM_021163894.1 | CCACCATGTACCCAGGCATT |
| *β-actin* R |  | AGGGTGTAAAACGCAGCTCA |

**Supplementary table S2. Antibodies used for IF**

| **Antibodies** | **Source** | **Identifier** |
| --- | --- | --- |
| Rabbit monoclonal anti-LC3B antibody | Abcam | Cat# ab192890 |
| Rabbit polyclonal anti-NCOA4 antibody | Proteintech | Cat# 10968-1-AP |
| Rabbit polyclonal anti-FTH1 antibody | Proteintech | Cat# 11682-1-AP |
| Goat anti-Rabbit IgG (H+L) Cross-Adsorbed Secondary Antibody, Alexa Fluo^TM^ 488 | ThermoFisher Scientific | Cat# A-11008 |
| Goat anti-Mouse IgG (H+L) Cross-Adsorbed Secondary Antibody, Cyanine3 | ThermoFisher Scientific | Cat# A10521 |

**Supplementary table S3. Bacteriostatic circle and MIC of *Aspergillus aureoles* GGNSJ001 against *S. aureus***

| **Names** | **Bacteriostatic Circle (mm)** | **MIC（μg/mL）** |
| --- | --- | --- |
| *Aspergillus aureoles* GGNSJ001 | 26 | 500 |
